# Physicochemical and Sensory Properties of Czech Lager Beers with Increasing Original Wort Extract Values during Cold Storage

**DOI:** 10.3390/foods11213389

**Published:** 2022-10-27

**Authors:** Richardos Nikolaos Salek, Eva Lorencová, Robert Gál, Vendula Kůrová, Kristýna Opustilová, František Buňka

**Affiliations:** 1 Department of Food Technology, Faculty of Technology, Tomas Bata University in Zlín, T.G. Masaryka 5555, 760 01 Zlín, Czech Republic; 2 Laboratory of Food Quality and Safety Research, Department of Logistics, Faculty of Military Leadership, University of Defence, Kounicova 65, 662 10 Brno, Czech Republic

**Keywords:** Czech beer, original wort extract, storage, physicochemical properties, sensory attributes

## Abstract

The scope of the study was the evaluation of the selected physicochemical (O_2_ and CO_2_ contents, bitterness, color, total polyphenol content (TPC), turbidity, foaming stability) and sensory properties of Czech lager beer with different original wort extract (OWE) values (OWE of 10.0; 11.0; 11.5; 12.0% *w*/*w*) during a cold storage period of 6 months (4 ± 2 °C). The length of the cold storage period did not influence the values of dissolved O_2_ and CO_2_, bitterness, color and foam stability of the samples. Contrarily, the TPC, turbidity, and sensory attributes of the samples were affected by the course of cold storage. The OWE values did not affect the development of the parameters tested. All beer samples stored until the 5th month presented “very good” sensory characteristics. Cold storage of beer is advantageous in order to maintain its freshness and sensory attributes at the highest level for the final consumer.

## 1. Introduction

Beer is a widely consumed carbonated alcoholic beverage around the world, being the first among alcoholic beverages [1,2,3]. The basic four raw materials in beer manufacture are water, barley (*Hordeum vulgare*) malt, hops (*Humulus lupulus* L.), and yeast. Furthermore, beer is produced through alcoholic fermentation by yeast that transforms sugars contained in malt wort mainly into ethanol, CO_2_, and other secondary metabolites (such as acetic, malic, succinic, lactic, and pyruvic acids) [4,5,6,7]. The styles of beer are numerous, and their diversification is based on different brewing techniques and ingredients utilized. In general, the most popular beer styles are lagers and ales, with lager being the most widely consumed and commercially available style of beer in the world. Lager beers are manufactured with the application of bottom-fermenting yeast strains (*Saccharomyces pastorianus*) fermented at low temperatures (usually in the range of 3.3–13.0 °C for a period from 4 to 12 weeks) [8,9,10]. 

Furthermore, Czech beer is a unique lager beer manufactured using high quality raw materials (barley malt, Saaz hops) together with the application of decoction mashing, bottom-fermentation, and long cold maturation (lagering). The term “Czech beer” is legally protected by the European Union Protected Geographical Indication (PGI). Typical characteristics of Czech beer include the presence of unfermented extract, higher bitterness and color values, higher polyphenol content, and higher pH. Furthermore, higher values of fullness and bitterness, longer bitterness fading, and lower presence of off-flavors are among the sensory attributes characterizing the unique type of beer mentioned above. Moreover, from the manufacturing technology point of view, (i) the composition of the applied wort (mainly influenced by the mashing strategy), (ii) the extent of hopping and (iii) the fermentation process used are the most important factors [11,12].

The original wort extract (OWE) value is the amount of material extracted from the wort (before fermentation) and is expressed in % (*w*/*w*). According to Czech legislation, beer can be divided into five categories based on the OWE value. In particular, (i) table beer (*stolní pivo*)—beer with an OWE of up to 6.00 % (*w*/*w*); (ii) draught beer (*výčepní pivo*)—beer with OWE values in the range of 7.00–10.99 % (*w*/*w*); (iii) lager beer (*ležák*)–bottom-fermented beer with OWE values between 11.00–12.99 % (*w*/*w*); (iv) full beer (*plné pivo*)—top-fermented beer with OWE values ranging from 11.00 to 12.99 % (*w*/*w*); and (v) strong beer (*silné pivo*)—beer with OWE values higher than 13.00 % (*w*/*w*). Furthermore, the latter classification of beer in the Czech Republic serves, among other things, for the calculation of the indirect tax on beer brewed by breweries (analogue of excise duty) [13]. The export of beer (in 2019) from the Czech Republic to the rest of the world was approximately 5.4 million hectoliters. However, increased export rates entail longer transport and storage times, leading to higher expectations regarding storage stability [14].

Beer is a chemically unstable product, and its composition continuously changes during storage, leading to gradual degradation in quality. The main factors destabilizing beer during storage are storage conditions (temperature, length, presence of light) and oxidation reactions. The storage temperature is the main parameter that contributes to the major changes in the chemical profile; the higher the storage temperature, the higher the rate of quality deterioration that might be expected [2,15,16].

Although the course of beer deterioration during storage is well documented, presenting significant quality changes after 3 to 6 months of storage at room temperature [17,18,19], the information available in the scientific literature about beer quality changes during a cold storage period is still scarce and is not given the appropriate attention in detail. The present work was carried out with the objective of evaluating selected physicochemical (O_2_ and CO_2_ contents, bitterness, color, total polyphenol content (TPC), turbidity, foaming stability) and sensory properties of Czech lager beer with different OWE (CB10.0—OWE of 10.0% *w*/*w*; CB11.0—OWE of 11.0% *w*/*w*; CB11.5—OWE of 11.5% *w*/*w*; CB12.0—OWE of 12.0% *w*/*w*) values during a 6-month cold storage (at 4 ± 2 °C) period.

## 2. Materials and Methods

### 2.1. Beer Sample Production

Four types of Czech lager beer with divergent OWE values were manufactured. The evaluated beers were manufactured under real industrial conditions (in a brewery located in the Olomouc Region, Czech Republic). The manufacture of the samples was designed to develop beer samples with a content of 10.00%, 11.00%, 11.50% and 12.00% (*w*/*w*) OWE and ethanol content within the interval of 4.00–5.00% (*v*/*v*). Typical technological processes commonly applied for the production of traditional Czech lager beers (brewing specifications are shown in Table 1) were utilized in the samples’ manufacture protocol. Three samples were manufactured without surrogates; the exception was the beer with 12.00% (*w*/*w*) of OWE, in which saccharose was applied. The fermentation process (6 ± 2 °C; for a period of 7 to 10 days according to the desired target OWE value) was realized in open vessels. The lagering of the beers was carried out in stainless steel vessels at 0.5 ± 1.0 °C for a period of time in the range of 50–60 days in the lager cellar. After lagering (cold maturation), beer samples were filtered (single-stage filtration with a desk filter) and stabilized with polyvinylpolypyrrolidone (PVPP; 600mg/L; SigmaAldrich, Prague, Czech Republic). Subsequently, the beers were stored in over-pressured tanks (0.54 ± 0.2% *w*/*w* CO_2_ content) for 24 h following packaging in brown glass bottles (volume of 0.5 l). Furthermore, the pasteurization process (25 pasteurization units (PU); 1 PU is defined as exposure to a temperature of 60 °C for 1 min)) was realized. The total volume of each production batch was 290 hl. The beers were stored for a period of 6 months in a controlled temperature chamber (at 4 ± 2 °C) in the absence of sunlight and UV radiation. Each beer sample was analyzed immediately after production (time 0) and after 30 days for a period of 6 months (*n =* 4 beer samples × 3 brewings for each sample × 6 months × 7 determinations × 6 repetitions *=* 1512 analyses performed in total).

### 2.2. Determination of Beer Ethanol, Density, Extract, Fermentation Degree and pH

The analyses were performed following standard Analytica European Brewery Convention (EBC) procedures [20]. Near-infrared spectroscopy was implemented using the Anton Paar Density Meter DMA 4500 M with the Alcolyzer Beer ME module (Anton Paar GmbH, Austria) to determine the ethanol content, density, apparent and real extract content, apparent and real degree of fermentation (EBC method No. 9.26) of the samples tested. Before analysis, the beer samples were degassed (EBC method No. 9.46) and then filtered on laboratory filter papers. Furthermore, the pH values (EBC method No. 9.35) of the beers were determined with a glass tip electrode of a calibrated pH meter (pH Spear, Eutech Instruments, Oakton, Malaysia). All measurements were performed at least six times (*n* = 6).

### 2.3. Determination of Beer Dissolved Carbon Dioxide and Oxygen Contents

The determination of the dissolved CO_2_ content of the beer samples was performed according to a physical method based on pressure measurement using a manometer (EBC method No. 9.28.3). The Haffmans InPack 2000 CO_2_-m (Pentair Südmo GmbH, Germany) was used for the development of pressure by mechanical shaking. The pressure and temperature of the samples were recorded (after stabilization). The Haffmans slide ruler was used to read the samples’ CO_2_ content (expressed as % *w*/*w*). The Haffmans Portable Optical CO_2_/O_2_/TPO Meter c-DGM (Pentair Südmo GmbH, Germany) was used for dissolved O_2_ content determination. EBC method No. 9.37.1 [20] was used, based on optical O_2_ sensors using the luminescence principle, where an O_2_-sensitive layer is illuminated by blue light, causing molecules in the O_2_-sensitive layer to enter an excited state. The concentration of O_2_ (expressed as mg/L ) was calculated from the temperature of the samples and the time elapsed between exposure and illumination. All measurements were performed at least six times (*n* = 6).

### 2.4. Determination of Beer Bitterness, Total Polyphenol Content, Color and Beer Foam Stability

Bitterness was determined following EBC method No 9.8 [20], with slight modifications using a UV-VIS spectrophotometer (DR 5000TM, HACH LANGE s.r.o, Czech Republic). In a centrifuge tube,10 mL of degassed beer sample, 0.5 mL of NHCl (SigmaAldrich, Ltd., Prague, Czech Republic) and 20 mL of isooctane (SigmaAldrich, Ltd., Prague, Czech Republic) were added. The tubes were shaken for 15 min and then centrifuged at 4032× *g* for 10 min at 20 ± 1 °C. The supernatant was measured at 275 nm against a blank control (pure isooctane). The IBU values were obtained as follows in Equation (1):

Bitterness (BU) = 50 × A275(1)

where: BU is bitterness units and A275 is absorbance at 275 nm.

The determination of TPC was performed according to EBC method No 9.1 using a UV-VIS spectrophotometer (DR 5000TM, HACH LANGE s.r.o, Czech Republic). Ten ml of degassed beer sample and 8 mL of CMC/EDTA reagent (carboxymethyl cellulose/ethylenediaminetetraacetic acid; SigmaAldrich, Ltd., Prague, Czech Republic) were transferred to a 25 mL volumetric flask and mixed. After mixing, 0.5 mL of ferric reagent (3.5% ammonium iron citrate; SigmaAldrich, Ltd., Prague, Czech Republic) was added to the sample, which was then thoroughly homogenized. Subsequently, 0.5 mL of ammonia reagent (ammonia:water, 1:2; SigmaAldrich, Ltd., Prague, Czech Republic) was added and thoroughly mixed. Finally, the volume was made up to 25 mL with distilled water and homogenized. The absorbance at 600 nm was measured after 10 min for the reaction to occur and stabilize. To obtain the content of polyphenols, the following Equation (2) was used:

TPC = A600 × 820(2)

where: TPC is total polyphenol content (mg/L ) and A600 is absorbance at 600 nm.

The color of the tested beer samples was determined according to EBC method No. 9.6 [20] using a UV-VIS spectrophotometer (DR 5000TM, HACH LANGE s.r.o, Czech Republic). Before analysis, the samples were centrifuged (at 3000× *g* for 5 min), and the collected supernatant was diluted in ultrapure water. The absorbance at 430 nm of the samples was determined against a blank of ultrapure water. The results were expressed as color units EBC (European Brewery Convention) according to the following Equation (3):

Color = A × f × 25(3)

where A is absorbance at 430nm and f is the dilution factor.

The determination of the stability of the beer foam was performed according to EBC method No. 9.42.1 [20] using the NIBEM-T foam stability tester (Haffmans BV, The Netherlands) device. The current instrument was used to measure the stability of beer foam (at 20 ± 1 °C) by measuring time intervals and monitoring the foam collapse time (s) by a certain distance (30 mm). In particular, the time period of drop of the foam–beer interface was measured. The beer samples were filled straight into the measuring vessel, which mimics the pouring of beer. The measurement started once the foam–beer interface dropped 10 mm under the edge of the cuvette. The results obtained were expressed as foam collapse time (FCT) in s.

### 2.5. Determination of Beer Turbidity (Haze)

The turbidity (suspended particulate concentration) of the examined beer samples was measured using the LABSCAT 2 (Sigrist-Photometer GmbH, Germany) dual-angle turbidimeter (in nephelometric mode, LED light source, wavelength 860 ± 30 nm) at 20 ± 1 °C according to EBC method No. 9.29 [20]. The angles were 90° and 25° in forward scattering. The samples prior to analysis were degassed in Erlenmeyer flasks by stirring (~2h). The results obtained were given in units of turbidity of the European Brewery Convention (EBC).

### 2.6. Sensory Analysis

The Czech lager beers evaluated in this study were subjected to sensory analysis, and parameters such as carbonation level, fullness, bitterness intensity, off-flavors and overall rating were determined. Definitions of the above-mentioned criteria are given by Rübsam, Gastl, and Becker [21]. Sensory analysis was performed by a panel of 16 selected assessors (expert) trained in describing beer according to ISO 8586-1 [22]. The assessors were between 24 and 61 years of age (9 women and 7 men) and were regular beer consumers (at least once a week). Beer samples were served in glasses (50 mL; coded with 3-digit numbers), odor-free, and covered by watch glasses. Samples were served in random order and at a controlled temperature of 20 ± 2 °C in a sensory laboratory equipped with sensory booths (each assessor was seated in a separate booth under normal light conditions) according to ISO 8586 [23]. Water and unsalted crackers were provided to cleanse the palate between samples, according to an appropriate washing procedure of 1 min. A 10 min break was taken after each sample to avoid fatigue of the palate. The beer samples were evaluated using 5-point scales for the following criteria: off-flavors, carbonation level, fullness, bitterness intensity (1—just recognizable, 3—moderate, 5—very strong). For the overall rating, a 9-point scale was used, where 1—extraordinarily good and 9—extremely bad.

### 2.7. Statistical Analysis

The physicochemical parameters examined were compared by analysis of variance (one-way ANOVA) followed by post-test (Tukey test), with 95% reliability. Data obtained were expressed as mean ± standard deviation. Additionally, the sensory properties of the beer samples were analyzed by non-parametrical analysis of variance of Kruskal–Wallis and Wilcoxon tests. The significance level used in the tests was 0.05. Statistical analyses were performed using Minitab^®^ 16 software (Minitab, Ltd., Coventry, UK).

## 3. Results and Discussion

### 3.1. Beer Ethanol, Density, Extract and pH

The basic physicochemical properties of the Czech lager beer samples with divergent OWE values are shown in Table 2. In general, significant statistical differences were observed among the evaluated beer samples for almost all basic physicochemical attributes examined (*p* ˂ 0.05). However, an exception was the pH value of the beers (*p* ≥ 0.05). From the results obtained, it could be stated that the brewing technology affected the basic physicochemical properties of the beers tested. In particular, the increasing levels of the OWE value resulted in beer samples with an increased ethanol content (*p* ˂ 0.05). An explanation for the above-mentioned statement could be found in the length of fermentation and the lagering period of the samples.

### 3.2. Beer Dissolved Carbon Dioxide and Oxygen Contents

**Figure 1:**
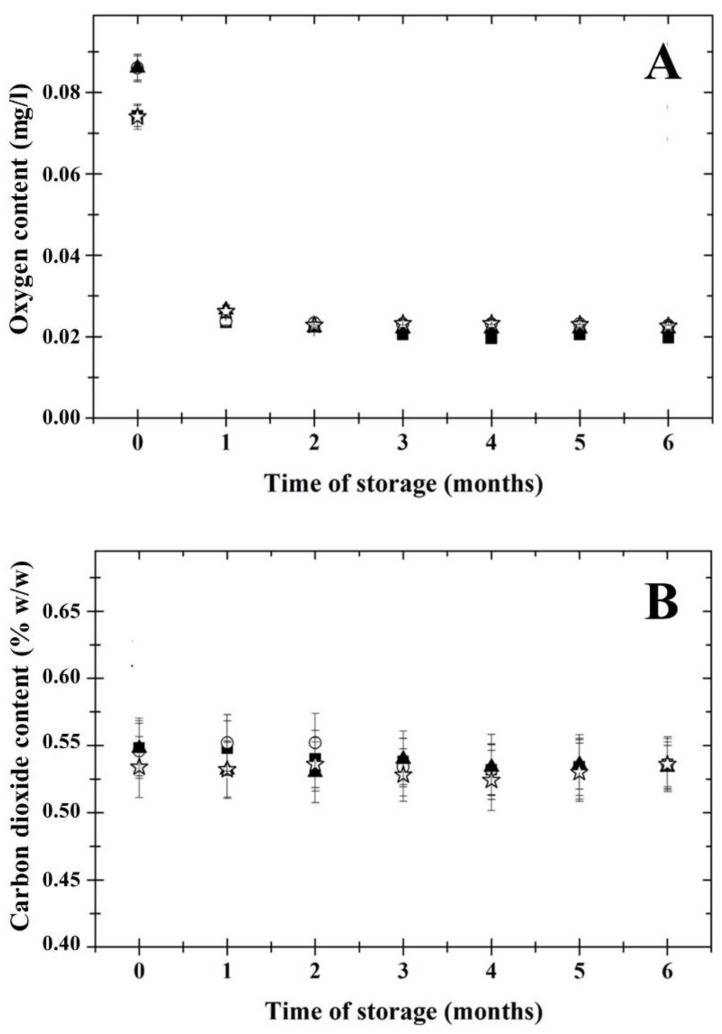
Development of CO_2_ content (% *w*/*w*; part **A**) and O_2_ content (mg/L; part **B**) in the Czech lager beer samples during a 6 months cold storage period at 4 ± 2°C. The examined beer samples presented different original wort extract values (■—10.0% *w*/*w*; ○—11.0% *w*/*w*; ▲—11.5% *w*/*w*; ☆—12.0% *w*/*w*). The results are expressed as means; the error bars represent standard deviation (*n* = 6).

Figure 1 shows the development of the dissolved CO_2_ and O_2_ contents in the evaluated Czech lager beer samples during a 6 months cold storage period (at 4 ± 2 °C). Oxygen content is a beer quality parameter, and one of the main objectives of the brewing industry is to maintain the O_2_ content at the lowest level throughout the manufacturing and storage period to avoid product deterioration and shelf-life shortening [10]. From the results obtained, it could be assumed that the length of cold storage did not affect the dissolved O_2_ content in the beer samples (*p* ˂ 0.05). Monitoring the dependence of the dissolved O_2_ content as affected by storage time proved to be important during the first month of cold storage. In particular, the highest amount of O_2_ was immediately after bottling (*p* ˂ 0.05), and after 1 month of storage, its amount decreased and remained almost constant throughout the storage time. A possible explanation for the reduction in the level of dissolved O_2_ could be the release of excess O_2_ into the bottle headspace [24]. The results are in accordance with those previously reported by Paternoster et al. [25]. Furthermore, according to Hempel et al. [10], the O_2_ content in beer should be below 0.1 mg/L. The dissolved O_2_ content in all tested Czech lager beer samples was in the range of 0.019–0.027 mg/L, over the last 5 months of cold storage.

Generally, the dissolved CO_2_ content in the evaluated beer samples was not influenced by the length of cold storage or the OWE value (*p* ≥ 0.05). According to Šulc, Bojas, and Dančová [26], the typical dissolved CO_2_ content for lager beers should be in the range of 0.4–0.5% (*w*/*w*). From the results illustrated in Figure 1, the tested beer samples presented CO_2_ content values approximately within the interval of 0.53–0.55% (*w*/*w*). The amount of CO_2_ in beer is a function of CO_2_ solubility, which in turn is affected by temperature, containing pressure, and beer composition [27]. From the results obtained, it is obvious that the storage of beer is an effective solution to maintain a dissolved CO_2_ content almost “constant” even for a period of 6 months. Overall, it is accepted that the amount of CO_2_ is one of the most important indicators of beer quality and organoleptic properties (sharpness).

### 3.3. Beer Bitterness, Total Polyphenol Content, Color and Foam Stability

**Figure 2:**
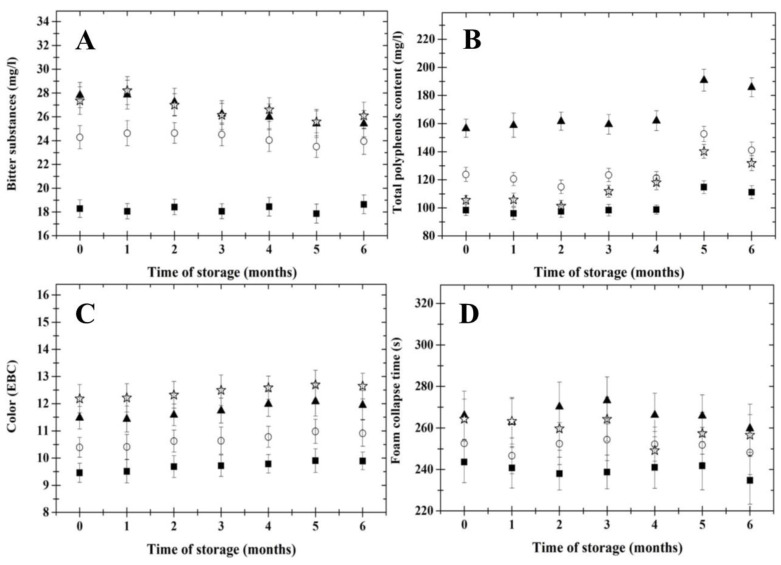
Development of bitter substances (mg/L; part **A**), total polyphenol content (mg/L; part **B**), color (EBC; part **C**) and foam collapse time (s; part **D**) of the Czech lager beer samples during a 6 months cold storage period at 4 ± 2 °C. The examined beer samples presented different original wort extract values (■—10.0% *w*/*w*; ○—11.0 % *w*/*w*; ▲—11.5% *w*/*w*; ☆—12.0% *w*/*w*). The results are expressed as means; the error bars represent standard deviation (*n* = 6).

The evolution of the bitterness of the beer samples during the 6 months cold storage period is depicted in Figure 2 (part A). In general, the bitterness of the tested samples was not affected by the length of the cold storage period (*p* ˂ 0.05). Bitterness values (immediately after bottling) for all samples tested were in the range of 18–28 (BU). The bitterness limit value for Czech beer has been determined as 22 EBC units [12]. All evaluated samples were in accordance with the latter limitation, with the exception of the sample CB10.0. Differences between the beer samples in terms of bitterness were probably due to divergent OWE values, which in turn could be affected by the brewing technology utilized (hopping strategy). Hops are one of the four basic ingredients in beer production; in addition to adding flavor and aroma, their main contribution to the finished beer is highly associated with the bitterness obtained during boiling of wort with hops. In particular, iso-α-acids, which are the products of the isomerization of α-bitter acids present in hops during the boiling of wort, are the main contributors to the bitterness of beer [28,29]. The highest bitterness values were observed for the CB11.5 and CB12.0 beer samples, respectively.

The TPC development of beer samples during cold storage is presented in Figure 2 (part B). From the results obtained, it could be reported that the OWE value of the samples and the length of cold storage affected the TPC of the monitored beers (*p* < 0.05). In particular, with the progress of storage time, the TPC of all samples increased. Significant changes in the samples’ TPC values were reported after the 4th month of storage. The development of TPC in the tested beer samples followed a similar course. According to Jurić et al. [30], the standard TPC values for lager beers are within the range of 50–150 mg/L. The TPC of the tested beer samples was in agreement with the interval mentioned above. Differences between TPC values in the samples might be due to divergent brewing techniques applied during the manufacture of the samples. The polyphenol content in beer differs with respect to the type of malt and hops used in the brewing process. About 80% of the polyphenols in beer originate from malt or adjuncts, and 20% originate from hops (*Humulus lupulus*). Phenolic compounds often appear in the form of esters and glycosides and may be bound to complex compounds, such as polysaccharides [31]. The compounds mentioned can directly influence beer quality and shelf-life. That is, polyphenols in beer can act as antioxidants, preventing oxidative degradation of beer while also providing potential health benefits to consumers through their inhibitory activity on certain mutagens and carcinogens. Additionally, polyphenols are diverse in chemical structure and can be divided into groups consisting of simple hydroxycinnamic and hydroxybenzoic acid derivatives (phenolic acids), flavanols, flavanol glycosides and prenylated flavonoids. In particular, flavanols are of great interest for brewers because they form protein–polyphenol complexes, leading to the formation of haze or turbidity in beer [32,33,34].

The evolution of the color of the beer samples during the 6 months cold storage period is shown in Figure 2 (part C). Generally, the length of cold storage did not influence the color of the samples (*p* ≥ 0.05). On the other hand, significant differences were observed between the samples in relation to their OWE value (*p* ˂ 0.05). Samples with higher OWE values reported increased values of color. A possible explanation for the observed color differences might be due to the divergent brewing techniques applied to the manufacture of the samples—in particular, the formation of melanoidins (such as furfural, hydroxymethylfurfural, and methylfurfural), which can be produced by Maillard reactions [2,3].

Foam stability was measured using the foam collapse time for Czech beer during cold storage (Figure 2; part D). The monitored values of foam collapse time for beer samples during cold storage were within the range of 235 to 270 s. The foam of a lager beer should be stable for a time period of approximately 200 s [27]. The foam stability was higher for beer samples with higher OWE values (*p* ˂ 0.05). Furthermore, the length of cold storage did not influence the foam stability of the samples (*p* ≥ 0.05). Factors influencing beer foam stability are: (i) high molecular malt proteins, (ii) interactions among malt proteins and isomerized α-bitter acids, (iii) viscosity and factors capable of decreasing surface tension (e.g., high fermentation temperatures, yeast autolysis, beer thermal treatment and storage temperature) [35]. Regarding our results, it could be stated that cold storage is an effective way to maintain the stability of beer foam over a period of 6 months. The findings mentioned above are important for the brewing industry because it is generally accepted that the presence of a stable and attractive foam head, especially for the style of lager beer, is an important criterion for determining beer quality [27].

### 3.4. Beer Foam Stability

**Figure 3:**
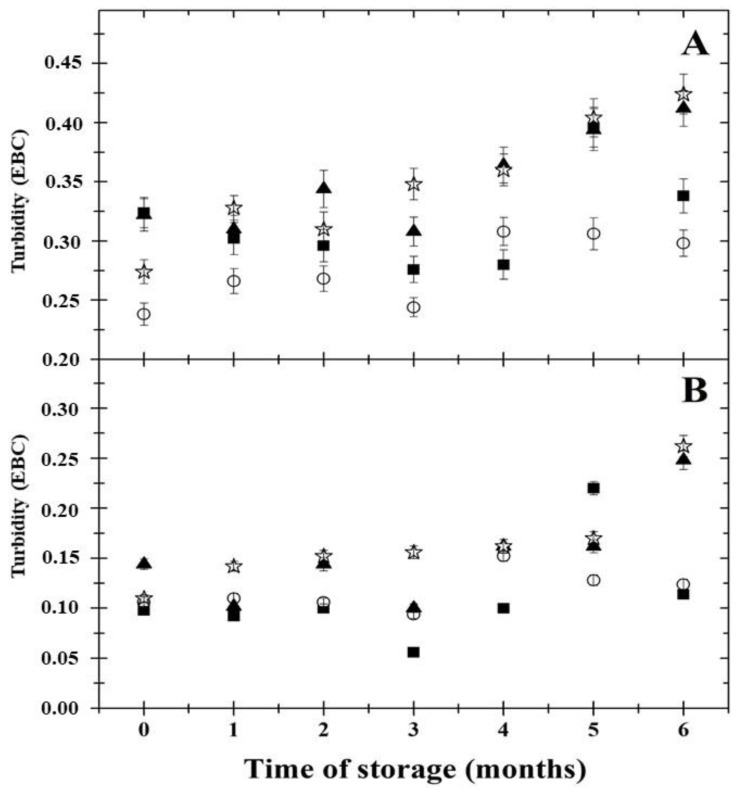
Development of turbidity values (EBC; 90° angle—part **A**; 25° angle—part **B**) of the Czech lager beer samples during a 6 months cold storage period at 4 ± 2°C. The examined beer samples presented different original wort extract values (■—10.0% *w*/*w*; ○—11.0% *w*/*w*; ▲—11.5% *w*/*w*; ☆—12.0% *w*/*w*). The results are expressed as means; the error bars represent standard deviation (*n* = 6).

Turbidity provides a visual sense of beer quality to consumers [35]. The results of the evolution of the beer turbidity values during the 6 months cold storage period are shown in Figure 3. From the obtained results, it could be stated that the OWE value of the samples and the length of cold storage affected the colloidal stability of the examined beers (*p* < 0.05). The highest turbidity values were reported for the CB11.5 and CB12.0 samples. According to Lorencová et al. [2], the 25° angle detects coarse haze particles larger than several micrometers, and the 90° angle determines colloidal haze. Beer turbidity increased during prolonged cold storage (Figure 3; parts A and B). Furthermore, the formation of larger particles was not significant (*p* ≥ 0.05); however, the exceptions were the samples CB11.5 and CB12.0 until the 5th month of storage, in which elevated values were reported (part B). On the other hand, the evolution of colloidal haze (90° angle; part A) was observed with the progress of cold storage for all tested samples. In particular, from the 4th month onward, increased turbidity values were detected. Furthermore, the monitored turbidity values (for both angles) did not exceed the specified limits for the brilliant visual assessment of beer. The limits mentioned above are 0.50 EBC for the 90° angle and 0.30 EBC for the 25° angle (EBC Method No. 9.29). The turbidity values increased with the increase in the OWE value throughout the storage period. The most frequent haze constituents in beer are proteins (containing proline) and polyphenols, which can bind together and form variably sized colloidal particles. Hordein (a barley prolamin; the main beer haze-active protein) with the action of polyphenols might lead to the formation of haze in beer. Furthermore, among other constituents of the development of colloidal haze in beer, polysaccharides, metal ions, hop resins and melanoidins have been reported [31,35,36]. A cold break (or chill) haze or permanent (or age-related) haze could be formed by interactions among proteins and polyphenols. Cold break haze forms at 0 °C and dissolves at higher temperatures. Contrarily, permanent haze forms initially when polypeptides and polyphenols are noncovalently bound; however, covalent bonds are soon formed, resulting in the formation of insoluble complexes. In addition, permanent haze is an irreversible process and could be affected by temperature, oxygen, heavy metals, agitation and light [31,35]. The statement above could be a possible explanation for the detected values of turbidity in the examined beer samples. It is generally accepted that the level of colloidal stability required for beer depends on the storage time and temperature after the beer has been bottled. Temperatures during transport can rise (in some cases ≥50 °C), leading to the formation of colloidal haze [36]. From the results obtained, it could be stated that it is essential to maintain beer at low temperatures during storage and transport.

### 3.5. Sensory Analysis

The results of the sensory analysis are shown in Table 3. Sensory analysis revealed that the length of cold storage affected all organoleptic attributes evaluated (*p* ˂ 0.05). All beer samples stored until the 5th month had “very good” organoleptic characteristics. In particular, panelists gave a similar rating for carbonation level, bitterness, and fullness until the 5th month of cold storage (*p* ≥ 0.05). The content of α-bitter acids that can transform into iso-α-bitter acids during wort hopping (boiling) and beer aging is the main reason for the emblematic bitter taste of lager beer [2]. According to Caballero et al. [37], the formation of iso-α-bitter acid degradation products over the course of beer cold storage can hardly be perceived. This statement is in agreement with the results in our study. However, after 5 months of cold storage, all beer samples presented significant signs of deterioration (*p* ˂ 0.05). Beer samples with OWE values ≥ 11.00% *w*/*w* showed a slight increase in off-flavors and received worse values in the overall rating attribute. The monitored increase in off-flavor of the samples could probably be attributed to cardboard flavor development, which is the major manifestation of beer staling [19]. Generally, chemical and flavor changes in beer during storage are mainly due to the development of sensory active substances (aldehydes, esters, and higher alcohols) arising from various steps of the brewing process [16]. However, more intensive “negative” sensory properties were observed in beer samples with values ≥11.00% *w*/*w*, probably due to the fact that the above-mentioned beers are “richer” in extractive substances (derived from barley malt and hops) [38]. The results of sensory analysis confirmed that cold storage could act as a “retarder” of beer flavor instability. That statement is in accordance with the findings of Ferreira et al. [16] and Heuberger et al. [39]. Furthermore, it is necessary to maintain low temperatures during the Czech lager beer storage and/or delivery chain to extend its shelf-life and thus retain unaltered its typical organoleptic properties for the longest possible time so that consumers all over the world can taste this unique type of beer in its full “freshness”. It is generally accepted that the consumer recognizes a brand of beer and associates it with a characteristic flavor profile. Therefore, it is essential for breweries to maintain the typical flavor of their products and to manufacture beers of constant quality over time [19,39].

## 4. Conclusions

The impact of the original wort extract (OWE) value on the selected physicochemical and sensory properties of beer samples during a cold storage period of 6 months was evaluated. In general, it could be concluded that the length of cold storage did not influence the values of dissolved O_2_ and CO_2_, bitterness, color, and foam stability of the evaluated beers. Contrarily, the total polyphenol content, turbidity and organoleptic attributes of the samples were affected by the course of cold storage. The intensity of the latter monitored changes did not indicate deterioration of the beer samples. In addition, the OWE values did not affect the development of the tested parameters; however, monitored differences could be attributed to divergent brewing techniques, resulting in various OWE values. All beer samples stored until the 5th month presented “very good” organoleptic characteristics. In general, from the obtained results, it could be stated that lower storage temperatures are advantageous for maintaining beer quality and shelf-life through the various stages of manufacture, storage, and distribution. Furthermore, the current study demonstrated the importance of cold storage of beer to maintain its freshness and sensorial attributes at the highest level for the final consumer (after storage and/or distribution). However, the elevated cost of cold storage should be mentioned as a limiting factor.

## Figures and Tables

**Table 1:** Brewing specifications.

	Values			
Mashing ^a^	CB10.0 **	CB11.0 **	CB11.5 **	CB12.0 **
Brew liquor/grist ratio	5.1/1 (L/kg)	5.1/1 (L/kg)	5.1/1 (L/kg)	5.1/1 (L/kg)
Mashing method	Double decoction process	Double decoction process	Double decoction process	Double decoction process
Mash-in temperature	40.0 (±2.0) °C	40.0 (±2.0) °C	40.0 (±2.0) °C	40.0 (±2.0) °C
pH of mashing	5.2 (±0.1)	5.2 (±0.1)	5.2 (±0.1)	5.2 (±0.1)
Mashing program	50 °C for 15 min	50 °C for 15 min	50 °C for 15 min	50 °C for 15 min
1st decoction (⅓ of the main mash)	65 °C for 55 min [from 65 °C to 97 °C (1 °C/min)]	65 °C for 55 min [from 65 °C to 97 °C (1 °C/min)]	65 °C for 55 min [from 65 °C to 97 °C (1 °C/min)]	65 °C for 55 min [from 65 °C to 97 °C (1 °C/min)]
Return to the main mash	65 °C for 45 min	65 °C for 45 min	65 °C for 45 min	65 °C for 45 min
2nd decoction (⅓ of the main mash)	70 °C for 60 min	70 °C for 60 min	70 °C for 60 min	70 °C for 60 min
Return to the main mash	80 °C for 60 min	80 °C for 60 min	80 °C for 60 min	80 °C for 60 min
Mash-out temperature	78 (±0.2) °C	78 (±0.2) °C	78 (±0.2) °C	78 (±0.2) °C
Total mashing time	267 min	267 min	267 min	267 min
**Hopping ^b^**				
**Hopping method**	Three-step hopping process	Three-step hopping process	Three-step hopping process (and extra dry hopping *)	Three-step hopping process
1st hopping	40% ^c^ out of the total quantity of α-bitter acids 5 min after the beginning of hop boiling	40% ^c^ out of the total quantity of α-bitter acids 5 min after the beginning of hop boiling	40% ^c^ out of the total quantity of α-bitter acids 5 min after the beginning of hop boiling	40% ^c^ out of the total quantity of α-bitter acids 5 min after the beginning of hop boiling
2nd hopping	30% ^c^ out of the total quantity of α-bitter acids 20 min after the beginning of hop boiling	30% ^c^ out of the total quantity of α-bitter acids 20 min after the beginning of hop boiling	30% ^c^ out of the total quantity of α-bitter acids 20 min after the beginning of hop boiling	30% ^c^ out of the total quantity of α-bitter acids 20 min after the beginning of hop boiling
3rd hopping	30% ^c^ out of the total quantity of α-bitter acids 15 min before the end of hop boiling	30% ^c^ out of the total quantity of α-bitter acids 15 min before the end of hop boiling	30% ^c^ out of the total quantity of α-bitter acids 15 min before the end of hop boiling	30% ^c^ out of the total quantity of α-bitter acids 15 min before the end of hop boiling
Total hopping time	80 min	80 min	90 min	120 min
**Fermentation**	6 days at 7 (±1) °C	7 days at 7 (±1) °C	9 days at 7 (±1) °C	10 days at 7 (±1) °C
Lagering (cold maturation) *	50 days 2 (±0.5) °C	55 days 2 (±0.5) °C	60 days 2 (±0.5) °C	60 days 2 (±0.5) °C

^a^ Czech malted barley (Pilsen-type malt from two-raw spring barley) was used in the manufacture of the beer samples. **^b^** The Saaz (semi-early red-bine) hops variety was applied; α-bitter acid content within the range of 3.0–4.5% *w*/*w*; β-bitter acid content within the range of 4.0–6.0% *w*/*w*; total polyphenol content in the range of 5.6–6.8% *w*/*w*. Overall dose of α-bitter acids was 3.5 g/L (for sample CB10.0); 5.5 g/L (for sample CB11.00); 7.0 g/L (for samples CB11.5 and CB12.0). **^c^** Total amount of α-bitter acids. * In the manufacture of CB11.5 beer sample, dry-hopping was applied 3 days before the end of cold maturation (day 57), hops were added in a level of 3 g/L. ** CB10.0 = 10.0% (*w*/*w*) original wort extract value; CB11.0 = 11.0% (*w*/*w*) original wort extract value; CB11.5 = 11.5% (*w*/*w*) original wort extract value; CB12.0 = 12.0% (*w*/*w*) original wort extract value.

**Table 2:** Basic physicochemical parameters of the evaluated Czech lager beer samples.

Parameter	Values			
	CB10.0 *	CB11.0 *	CB11.5 *	CB12.0 *
Ethanol				
% *v*/*v*	3.94 ± 0.02 ^a^	4.38 ± 0.01 ^b^	4.50 ± 0.02 ^c^	4.78 ± 0.01 ^d^
% *w*/*w*	3.09 ± 0.01 ^a^	3.43 ± 0.01 ^b^	3.52 ± 0.03 ^c^	3.74 ± 0.01 ^d^
Extract of original wort (% *w*/*w*)	9.88 ± 0.09 ^a^	10.89 ± 0.05 ^b^	11.44 ± 0.08 ^c^	11.85 ± 0.07 ^d^
Extract				
Apparent (% *w*/*w*)	2.26 ± 0.04 ^a^	2.51 ± 0.02 ^b^	2.76 ± 0.02 ^c^	2.91 ± 0.01 ^d^
Real (% *w*/*w*)	3.71 ± 0.04 ^a^	4.10 ± 0.03 ^b^	4.39 ± 0.02 ^c^	4.63 ± 0.01 ^d^
Fermentation degree				
Apparent (% *w*/*w*)	76.71 ± 0.15 ^a^	77.19 ± 0.11 ^b^	77.68 ± 0.17 ^c^	78.13 ± 0.14 ^d^
Real (% *w*/*w*)	61.01 ± 0.16 ^a^	61.57 ± 0.12 ^b^	62.31 ± 0.17 ^c^	62.69 ± 0.15 ^d^
Color (EBC Units)	9.46 ± 0.05 ^a^	10.39 ± 0.03 ^b^	12.21 ± 0.04 ^c^	11.53 ± 0.02 ^d^
Density (g/L)	1.041 ± 0.05 ^a^	1.044 ± 0.03 ^b^	1.046 ± 0.04 ^c^	1.048 ± 0.05 ^d^
pH (−)	4.55 ± 0.01 ^a^	4.52 ± 0.02 ^a^	4.51 ± 0.01 ^a^	4.54 ± 0.01 ^a^

* CB10.0 = 10.0% (*w*/*w*) original wort extract value; CB11.0 = 11.0% (*w*/*w*) original wort extract value; CB11.5 = 1.5% (*w*/*w*) original wort extract value; CB12.0 = 12.0% (*w*/*w*) original wort extract value. The mean values within a row (the difference between the beer samples) followed by different superscript letters statistically differ (*p* < 0.05).

**Table 3:** Sensory attributes of the beer samples (carbonation level, fullness, bitterness intensity, off-flavors and overall rating).

Beer Samples ^2^	Storage Time (Months)	Sensory Evaluation ^1^				
		Carbonation Level ^3^	Fullness ^3^	Bitterness Intensity ^3^	Off-Flavors ^3^	Overall Rating ^3^
CB10.0	0	3 ^aA^	3 ^aA^	3 ^aA^	2 ^aA^	3 ^aA^
	1	3 ^aA^	3 ^aA^	3 ^aA^	1 ^bB^	3 ^aA^
	2	3 ^aA^	3 ^aA^	3 ^aA^	1 ^bB^	3 ^aA^
	3	3 ^aA^	3 ^aA^	3 ^aA^	3 ^cC^	4 ^bC^
	4	3 ^aA^	3 ^aA^	3 ^aA^	2 ^aA^	4 ^bC^
	5	2 ^bC^	2 ^aC^	3 ^aA^	3 ^cC^	4 ^bC^
	6	2 ^bC^	2 ^aC^	3 ^aA^	3 ^cC^	5 ^bD^
CB11.0	0	3 ^aA^	3 ^aA^	3 ^aA^	1 ^bB^	3 ^aA^
	1	3 ^aA^	3 ^aA^	4 ^bB^	1 ^bB^	3 ^aA^
	2	3 ^aA^	3 ^aA^	3 ^aA^	2 ^aA^	4 ^bC^
	3	3 ^aA^	3 ^aA^	3 ^aA^	2 ^aA^	4 ^bC^
	4	3 ^aA^	3 ^aA^	3 ^aA^	3 ^cC^	4 ^bC^
	5	2 ^bC^	2 ^aC^	3 ^aA^	3 ^cC^	5 ^cD^
	6	2 ^bC^	2 ^bC^	3 ^aA^	4 ^dD^	5 ^cD^
CB11.5	0	4 ^cB^	3 ^aA^	3 ^aA^	1 ^bB^	2 ^dB^
	1	3 ^aA^	3 ^aA^	3 ^aA^	1 ^bB^	2 ^dB^
	2	3 ^aA^	3 ^aA^	3 ^aA^	3 ^cC^	4 ^bC^
	3	3 ^aA^	3 ^aA^	3 ^aA^	3 ^cC^	4 ^cC^
	4	3 ^aA^	3 ^aA^	4 ^bB^	3 ^cC^	4 ^bC^
	5	2 ^bC^	2 ^aC^	3 ^aA^	3 ^cC^	5 ^cD^
	6	2 ^bC^	2 ^bC^	3 ^aA^	4 ^dD^	6 ^eE^
CB12.0	0	3 ^aA^	4 ^cB^	3 ^aA^	1 ^bB^	2 ^dB^
	1	3 ^aA^	3 ^aA^	3 ^aA^	1 ^bB^	2 ^dB^
	2	3 ^aA^	3 ^aA^	3 ^aA^	2 ^aA^	4 ^bC^
	3	3 ^aA^	3 ^aA^	3 ^aA^	2 ^aA^	4 ^bC^
	4	3 ^aA^	3 ^aA^	3 ^aA^	3 ^cC^	4 ^bC^
	5	2 ^bC^	2 ^aC^	3 ^aA^	3 ^cC^	5 ^bD^
	6	2 ^bC^	2 ^aC^	3 ^aA^	4 ^cD^	6 ^cE^

^1^ The median values within a column (the difference between the storage times) followed by different superscript letters statistically differ (*p* < 0.05); the samples manufactured with different extract or original wort value were evaluated independently. The median values within a column (the difference between the original wort extract values) followed by different capital letters statistically differ (*p* < 0.05); the samples stored for different times were evaluated independently. ^2^ CB10.0 = 10.0% (*w*/*w*) original wort extract value; CB11.0 = 11.0% (*w*/*w*) original wort extract value; CB11.5 = 11.5% (*w*/*w*) original wort extract value; CB12.0 = 12.0% (*w*/*w*) original wort extract value. ^3^ Carbonation level: 1—just recognizable; 3—moderate; 5—very strong. Fullness: 1—just recognizable; 3—moderate; 5—very strong. Bitterness intensity: 1—just recognizable; 3—moderate; 5—very strong. Off-flavors: 1—just recognizable; 3—moderate; 5—very strong. Overall rating: 1—extraordinarily good; 9—extremely bad.

## Data Availability

The data presented in this study are available on request from the corresponding author.
